# Exploring the Association of Fear, Mental Health, and Lifestyle with Insomnia: A Machine Learning Approach among University Students

**DOI:** 10.1055/s-0046-1829077

**Published:** 2026-11-06

**Authors:** Sara Khademi, Mohammad Reza Saebipour, Hamid Saadatfar, Seyed Mohammad Riahi

**Affiliations:** 1 Department of Pediatrics, Student Research Committee, Birjand University of Medical Sciences, BirjandIran; 2 Department of Anatomy, Birjand University of Medical Sciences, BirjandIran; 3 Department of Computer Engineering, Faculty of Electrical and Computer Engineering, University of Birjand, BirjandIran; 4 Department of Family and Community Medicine, School of Medicine, Cardiovascular Diseases Research Center, Birjand University of Medical Sciences, BirjandIran

**Keywords:** insomnia, fear, mental health, lifestyle, university students

## Abstract

**Objective:**

The aim of this study was to identify key predictors of insomnia among university students, focusing on psychological, behavioral, and lifestyle factors. The study also aimed to explore the role of machine learning in identifying insomnia-related patterns and informing personalized intervention strategies.

**Methods:**

A cross-sectional study was conducted with 576 students from universities in Birjand during the 2023–2024 academic year. Machine learning methods, including Random Forest, Decision Tree, and SHAP analysis, were used for feature importance analysis and knowledge extraction.

**Results:**

Self-reported questionnaires revealed that fear of not falling asleep particularly in the late hours and stress were the most significant predictors of insomnia. Anxiety, poor sleep hygiene, and depression were also strongly associated with insomnia. Other associated factors included financial and family concerns, bedtime, wake-up time, sleep duration, and social media usage. Machine learning models further highlighted these factors, with those showing poor sleep hygiene and high anxiety levels being more likely to experience insomnia.

**Discussion:**

The findings underscore the complex relationship between mental health issues and sleep disorders, particularly insomnia, in university students. The results also suggest a bidirectional relationship between insomnia, anxiety, depression, and stress. The use of machine learning showed potential for identifying insomnia-related patterns and informing personalized intervention strategies. These results highlight the need for effective strategies to address insomnia in students, particularly in light of the growing stressors faced in academic and personal life. However, due to the cross-sectional design, causal inferences cannot be drawn.

## Introduction

Quality and sufficient sleep are fundamental daily needs for humans. Studies suggest that the optimal sleep duration for cognitive function is 6 to 7 hours. 1 Various factors, including physical, emotional, and psychological conditions, can disrupt sleep patterns and lead to sleep disorders. Insufficient sleep can severely impact both physical and mental health, significantly reducing daily performance. 2

Insomnia is one of the most common sleep disorders, characterized by dissatisfaction with sleep quality or quantity, accompanied by difficulty initiating sleep, maintaining sleep continuity, early morning awakening, and inability to return to sleep. 3 The prevalence of insomnia has been reported to range from 5% to 50%. 2 Factors such as aging, unemployment, poor socioeconomic status, lifestyle, and poor sleep hygiene increase the risk of insomnia. Moreover, chronic insomnia can lead to serious health consequences and even elevate the risk of mortality. 4 Chronic insomnia is associated with a heightened risk of developing migraines, 5 Alzheimer's disease, 6 and cardiovascular diseases. 7

Effective sleep is particularly critical for university students, as it not only influences their health but also their academic performance. 8 However, the living conditions of students make them especially vulnerable to insomnia, with its prevalence in this population reported to be significantly higher than in the general population. 9 Consequently, investigating insomnia among students has gained considerable attention in recent years.

As noted, various factors contribute to insomnia and increase its likelihood among students. According to previous studies, 10 11 12 stress, anxiety, and depression are critical contributors to insomnia in this demographic. Additionally, studies 13 14 15 have identified sleep hygiene as a significant factor influencing insomnia. This factor is particularly relevant for students, given their living environments and the challenges in maintaining a controlled sleep environment and a regular sleep-wake schedule. Fear of insomnia is another sleep-related factor that has been highlighted in earlier research. 16 However, this factor may warrant further investigation given its strong association with insomnia.

Given the importance of sleep for overall health and its profound effects on the quality of life and performance, especially among students, it is essential to investigate insomnia in this group. Moreover, given the potential of machine learning techniques to analyze complex datasets comprehensively, the present study employs machine learning methods to examine the contributing factors to insomnia in students.

## Methods

This cross-sectional study was conducted during the 2023-2024 academic year among students enrolled at universities in Birjand, Iran. A total of 576 participants were selected using stratified random sampling. Participants were fully informed about the objectives of the study, and informed consent was obtained from all participants. The inclusion criteria required participants to be current students in Birjand and willing to participate in the study. Exclusion criteria included unwillingness to participate, pregnancy, and being diagnosed with or undergoing treatment for cancer.

### Data Collection Tools

The students were asked to complete a self-administered questionnaire. This questionnaire included a checklist covering demographic information, physical activity, social media activity, hours spent using digital devices, history of underlying diseases and medication use, caffeine consumption, and routine sleep habits. Additionally, participants completed three validated questionnaires: the Insomnia Severity Index (ISI), the Sleep Hygiene Index (SHI), and the Depression, Anxiety, and Stress Scale (DASS). These instruments were used to comprehensively evaluate factors contributing to insomnia and related psychological and behavioral aspects.

#### Insomnia Severity Index (ISI) Questionnaire

The Insomnia Severity Index (ISI) is a standardized questionnaire comprising seven items that evaluate an individual's perception of their insomnia. Each item is rated on a five-point Likert scale, with higher scores indicating more severe insomnia. The total score ranges from 0 to 28. In the present study, individuals with a score of 15 or above were categorized as having clinical insomnia, with 15 as the cutoff. 17 The Persian version of the ISI has been validated and shown to be reliable in previous research. 18

#### Sleep Hygiene Index (SHI) Questionnaire

The Sleep Hygiene Index (SHI) includes 13 items in which participants rate the frequency of certain behaviors on a five-point scale: always, often, sometimes, rarely, or never. Scores on this index range from 0 to 52, with higher scores reflecting poorer sleep hygiene. The Persian version of this tool has been validated and shown to be reliable in previous research. 19

#### Depression, Anxiety, and Stress Scale (DASS-21) Questionnaire

The Depression, Anxiety, and Stress Scale (DASS) is a set of three self-report subscales designed to measure emotional states of depression, anxiety, and stress. The original scale consists of 42 items; however, the short-form version (DASS-21), which includes 21 items, was used in this study. Each subscale comprises seven questions. The validity and reliability of the Persian version of this questionnaire have been evaluated and confirmed in previous studies. 20

### Data Analysis Tools for Feature Selection

To identify the key features associated with insomnia, a combination of machine learning methods was employed, including Decision Tree, Random Forest, and SHAP (SHapley Additive exPlanations) analysis. These approaches were utilized for feature selection and to provide a comprehensive understanding of the factors contributing to insomnia. Below is a brief description of each method and its application in this study

- Decision Tree is a supervised learning method widely used for both classification and regression tasks. Its hierarchical structure enables efficient training and inference with logarithmic time complexity. The interpretability of decision trees makes them suitable for identifying key features, as the splits at each node represent important thresholds for the dataset. Decision trees were used to identify initial factors associated with insomnia by analyzing the dataset and determining significant feature splits.
- Random Forest is an ensemble learning method that aggregates predictions from multiple decision trees to improve model performance and reduce overfitting. Each tree is trained independently, and the final output is obtained by combining their predictions. Random Forest provides a ranking of feature importance based on the contribution of each feature across all trees in the ensemble. Random Forest was employed to further validate and refine the factors identified by the decision tree, ensuring robustness and reliability in feature selection.
- SHAP is a game-theory-based method for interpreting the predictions of machine learning models. It attributes the output of the model to its input features, quantifying the contribution of each feature to the prediction. SHAP analysis provides a deeper understanding of the role and impact of each feature. SHAP was used to conduct a more comprehensive analysis of the factors associated with insomnia, offering detailed insights into how specific features affect the prediction outcomes.
- By combining these methods, the key factors contributing to insomnia in the study population were systematically identified and analyzed. The integration of SHAP with Decision Tree and Random Forest enabled a more accurate and interpretable feature selection process, providing robust results for understanding insomnia-related factors.

### Data Analysis Tools for Classification

In addition to Decision Tree and Random Forest, four other machine learning methods were employed to classify individuals with insomnia from healthy individuals based on input features: K-Nearest Neighbors (KNN), Logistic Regression, Naive Bayes, and Multi-Layer Perceptron (MLP). The performance of these models was evaluated using precision, recall, accuracy, and F1-score to ensure a comprehensive assessment.

- K-Nearest Neighbors (KNN) is a non-parametric method that classifies data based on the majority class among the k-nearest neighbors. It assumes that similar instances are found in close proximity, making it useful for pattern recognition.
- Logistic Regression is a statistical model used for binary classification. It estimates the probability of a binary outcome and uses a logistic function to map predicted values to a range between 0 and 1.
- Naive Bayes is a probabilistic classifier based on Bayes' theorem, assuming the independence of features. It computes the posterior probability for each class and selects the class with the highest probability.
- Multi-Layer Perceptron (MLP) is a type of artificial neural network with multiple layers of nodes, capable of modeling complex relationships in data. It uses backpropagation to adjust weights and improve prediction accuracy.

A 5-fold cross-validation approach was used for all evaluations to provide reliable and unbiased performance metrics. Given the imbalance between the two classes (insomnia and healthy individuals), cost-sensitive learning was applied, assigning a weight of 5 to the insomnia class and 1 to the healthy class.

To ensure that all features contributed equally to the analysis and prevent any feature from dominating the model due to differences in scale, the data were standardized using the Min-Max scaling method. This method transformed the data to a uniform range, improving model performance and consistency across different features.

This multi-method approach, combined with robust evaluation metrics and techniques, enabled a thorough and balanced analysis of the classification models for identifying individuals with insomnia.

## Results

A total of 576 university students in Birjand were included in the final analysis; 3 participants were excluded due to pregnancy during data collection and were not included in this final sample. The mean age of the participants was 21.5 ±2.99 years, with 60.9% female and 39.1% male participants. The mean BMI was 21.9 ± 3.81 kg/m2. Regarding insomnia severity, 34.7% of participants scored between 0–7 (minimal or no insomnia), 47.9% scored between 8–14 (subthreshold insomnia), and 17.4% scored 15 or above (indicating clinical insomnia).

All participants were categorized into two classes based on their ISI scores: Class 1 includes samples with scores ranging from 0 to 14, while Class 2 consists of samples with scores greater than or equal to 15. To identify the factors associated with the distinction between healthy individuals and those with insomnia disorder, we employ machine learning-based feature selection methods. These methods can provide a comprehensive view of the importance of each feature based on their impact on the learning process. Feature importance was evaluated using two methods: decision trees and random forests. It is important to note that, due to the imbalance between the classes (476 samples in Class 1 and 100 samples in Class 2), cost-sensitive versions of decision trees and random forests (with a 1:5 ratio) were used in the analysis. Additionally, to perform the learning process and evaluate its performance in all experiments, K-fold cross-validation with K = 5 was applied.

Fig. 1.A shows the feature importance calculated using the decision tree method, based on the average decrease in impurity, measured by the (Gini index). Fear of not falling asleep, stress, anxiety, sleep hygiene (SHI score), and sleep duration were the five factors with the greatest importance in distinguishing between the two classes. These results are largely consistent with those from the random forest method Fig. 1.B .

**Fig. 1:**
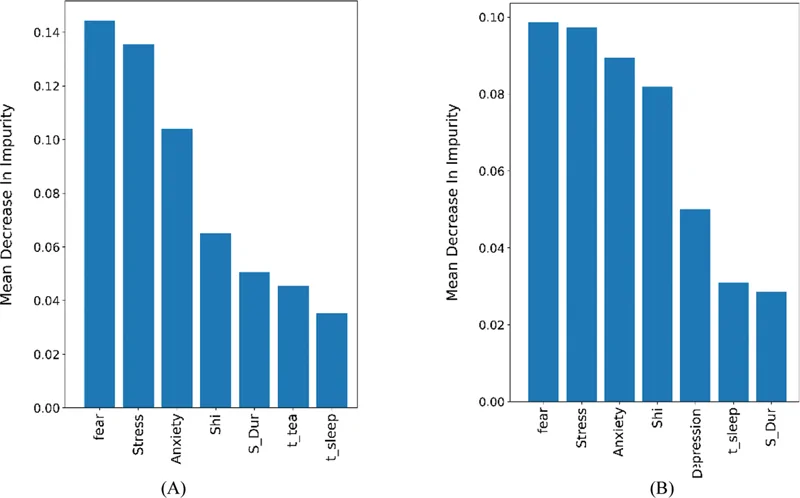
(A) Feature importance based on decision tree (B) Feature importance based on random forest. **Abbreviations:** Shi, sleep hygiene index; S_Dur, sleep duration; t_sleep, sleep onset time relative to the 12:00 midnight reference point; t_tea, time elapsed since the last tea consumption.

To more effectively assess the relationship between various parameters and the insomnia severity index, an efficient, novel, and unique method known as SHAP has been utilized. A high degree of consistency was observed between the most important features from the SHAP analysis and those identified by the decision tree and random forest methods, supporting the reliability of the findings. Fig. 2 illustrates the most important features based on the average SHAP value for each feature. Higher SHAP values correspond to greater importance of the feature. As seen, fear of not falling asleep and stress have been identified as the two most significant factors, with anxiety and sleep hygiene being the next most important features from the SHAP analysis perspective.

**Fig. 2:**
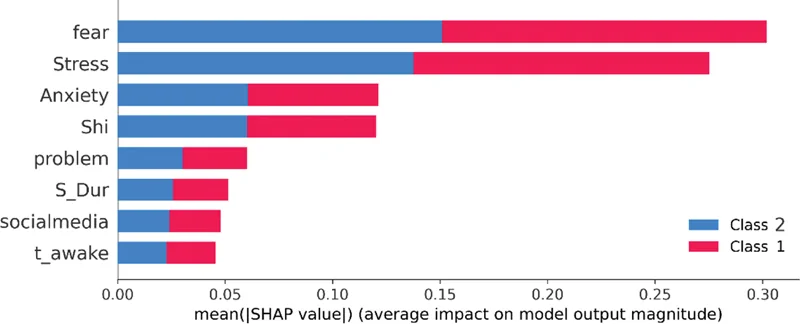
The most important features based on the mean SHAP value. **Abbreviations:** Shi, sleep hygiene index; S_Dur, sleep duration; t_awake, wake-up time relative to the 12:00 midnight reference point.

As illustrated in Fig. 3 , an increase in the levels of fear of not falling asleep, stress, anxiety, and reduced sleep hygiene (higher values of SHI) is directly associated with a higher likelihood of being classified as part of the patient group. Conversely, the absence of occupational, financial, and familial concerns was associated with a higher likelihood of classification within the healthy group. Additionally, a higher number of active social networks and shorter sleep duration are directly correlated with the classification of individuals into the patient group.

**Fig. 3:**
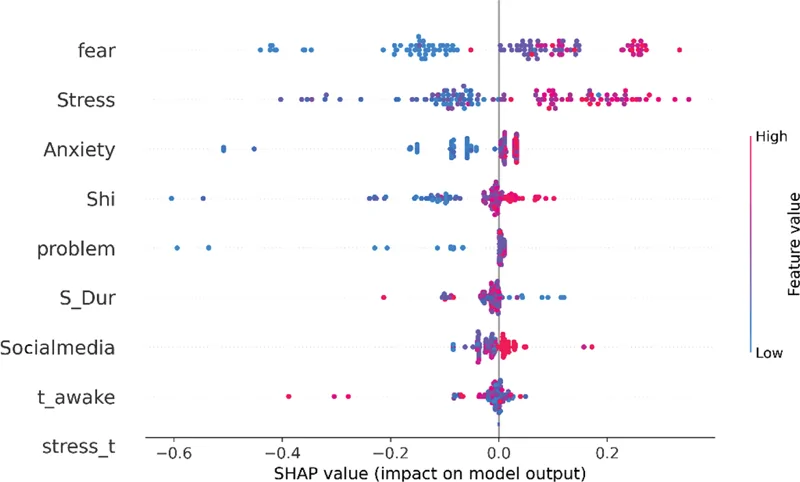
SHAP summary plot showing how feature values affect the model output. **Abbreviations:** Shi, sleep hygiene index; S_Dur, sleep duration; t_awake, wake-up time relative to the 12:00 midnight reference point; stress_t, time elapsed since the last stressful event.

In Fig. 3 , red indicates higher feature values, while blue represents lower values. Furthermore, the greater the SHAP value for a specific feature, the greater its contribution to the classification of individuals into the patient group.

Table 1 presents the performance of the classification models in the binary classification task. The evaluation of the models was conducted using the k-fold cross-validation method with k = 5. Due to the class imbalance in the binary classification problem, cost-sensitive methods were employed with weights of 1 and 5 assigned to the healthy class and the insomnia class, respectively. The Random Forest model demonstrated the best overall performance. These models showed potential for predicting insomnia status among students.

**Table 1:** Performance of Different Machine Learning Models in Binary Classification of Insomnia

Indicators	Precision		Recall		Accuracy	F1-score
Models	Class 1	Class 2	Class 1	Class 2		
Decision Tree	0.87	0.33	0.82	0.43	0.75	0.76
Random Forest	0.88	0.46	0.89	0.43	0.81	0.81
Logistic Regression	0.90	0.36	0.77	0.61	0.75	0.77
Naïve Bayes	0.91	0.41	0.82	0.61	0.78	0.80
K-Nearest Neighbor (KNN)	0.83	0.37	0.97	0.55	0.82	0.76
Multi-Layer Perceptron (MLP)	0.86	0.39	0.89	0.31	0.79	0.79

Each node of the decision tree shown in Fig. 4 represents a feature (e.g., fear, life problems, SHI score), with thresholds guiding splits. Leaves display total samples (left) and misclassified samples (right), indicating class probabilities at each endpoint. As can be seen in Fig. 4 , the decision tree analysis highlights key factors associated with the likelihood of insomnia. Higher levels of fear, greater number of life problems (e.g., family, financial, or academic issues) and poor sleep hygiene index (Shi > 20) are associated with a higher probability of belonging to the insomnia group. Shorter sleep durations (S_Dur ≤ 7.25 hours) and elevated stress levels are also associated with a higher likelihood of insomnia. Conversely, lower stress, longer sleep duration, and the absence of major personal, financial, or occupational problems are associated with a lower likelihood of insomnia, increasing the likelihood of being classified as healthy. These findings highlight the potential relevance of psychological stressors and sleep hygiene in understanding insomnia among university students.

**Fig. 4:**
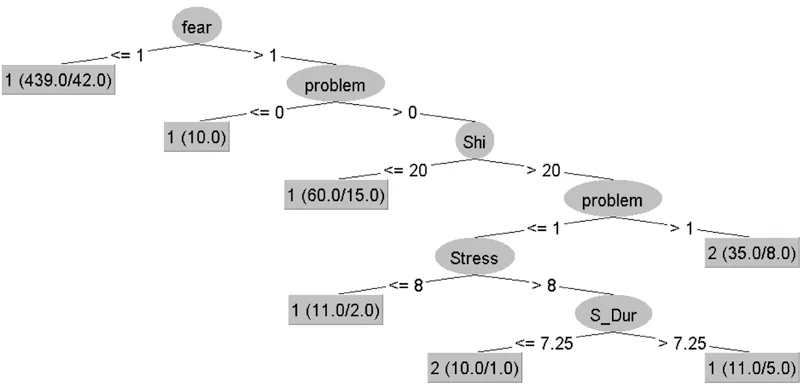
Decision tree and classification rules for distinguishing insomnia cases from healthy controls. **Abbreviations:** Shi, sleep hygiene index; S_Dur, sleep duration.

If a student reports never or rarely experiencing fear of falling asleep, they have approximately a 0.9 probability of being classified as healthy. For those who report higher levels of fear but maintain good sleep hygiene (Shi ≤ 20), the probability of being classified as healthy decreases to 0.75. However, if they also fail to maintain good sleep hygiene and face significant life problems, they are 77% likely to be classified in the insomnia group. These findings highlight the combined association of psychological factors, sleep hygiene, and life challenges with insomnia risk.

## Discussion

The present study was conducted to investigate the factors associated with insomnia among students. A total of 576 students from universities in Birjand during the 2023–2024 academic year participated in the study. The mean age of the participants was 21.5 ± 2.99 years, with 60.9% of them being female and 39.1% male. The findings showed that 34.7% of the participants had an insomnia severity index (ISI) score of 0–7, 47.9% had an ISI score of 8–14, and 17.4% had an ISI score of 15 or higher. These results align with a systematic review conducted in 2015 on the prevalence of insomnia among students. 9 Previous studies have reported the prevalence of insomnia among students to range between 14% and 47%. 21

For instance, a study conducted in 2022 on 856 students at Cumhuriyet University in Turkey reported an insomnia prevalence of 12.1%. 22 Similarly, a 2020 study on 669 students at Jazan University in Saudi Arabia reported an insomnia prevalence of 19.3%. 12 Another study by Moayedy and colleagues in 2015, conducted on medical students at Hormozgan University of Medical Sciences, reported an insomnia prevalence of 45.3%. 23

The variations in insomnia prevalence reported in recent studies may be attributed to differences in insomnia assessment tools, participants' age, academic terms, fields of study, associated academic and career concerns, target populations, and cultural differences. Moreover, sampling during examination periods could significantly influence reported prevalence rates. For example, in a 2022 study by Carrión-Pantoja et al., the prevalence of insomnia among students who responded during exam periods was significantly higher at 52.2%. 14

According to the results of the present study, fear of not falling asleep was identified as the most significant predictor of insomnia disorder. There is a subtle distinction between the fear of not being able to sleep and anxiety as broadly defined. Of course, a precise distinction between the two will have practical implications for diagnosis and intervention. Previous studies have discussed fear and anxiety during sleep onset due to various reasons. For instance, one study evaluated concerns about not sleeping, including fear of potential risks during sleep, using machine learning techniques. This study reported the negative impact of such fears on sleep quality. 24 However, despite acknowledgment of the importance of fear of not falling asleep in the literature, few studies have specifically investigated this factor. 25

Fear of not falling asleep late at night or upon going to bed—stemming from prior experiences of sleeplessness and subsequent anxiety about the consequences of insufficient sleep and impaired performance the following day—can lead to cognitive arousal. Since sleep typically does not occur during periods of heightened cognitive arousal, this fear may exacerbate insomnia disorder. 16 26 Thus, fear of not falling asleep may represent an important factor associated with insomnia and may warrant further investigation as a potential target for future interventions.

Sleep hygiene is another critical factor associated with insomnia among students, as extensively demonstrated in the literature. Adherence to sleep hygiene principles has been recognized as more important than many other interventions and has even led to the development of clinical guidelines.

In a 2022 study conducted on students in Spain, sleep hygiene was reported as one of the strongest independent predictors of insomnia. 14 Similarly, a study by Manzar and colleagues in 2020 on 525 students at Mizan-Tepi University in Ethiopia found that poor sleep hygiene was associated with difficulty initiating sleep, frequent awakenings, and impaired daytime functioning. 13

As the results suggest (e.g., the decision tree in Fig. 4 ), adherence to sleep hygiene principles was associated with a lower likelihood of insomnia in individuals reporting a fear of not falling asleep. Given that certain sleep hygiene practices—such as maintaining a consistent sleep and wake schedule, avoiding digital devices in bed, refraining from caffeine and smoking close to bedtime, avoiding exercise near bedtime, and controlling room lighting and noise—are easier to implement and have fewer side effects compared with some pharmacological interventions, sleep hygiene may represent an important factor to consider in understanding and addressing insomnia among students. This is unsurprising, as numerous studies have emphasized the importance of sleep hygiene over other interventions, which has led to its inclusion in clinical guidelines. On the other hand, student life – including exam stress and shared dormitories – disrupts routines and limits sleep hygiene control, highlighting the potential value of further research on tailored interventions in this population. 27

Global studies conducted on diverse student populations have identified a moderate to high prevalence of depression, anxiety, and stress within this group. 28 Students face various stress-inducing circumstances, including being away from family, adapting to university environments, interacting with new individuals, changes in course content and teaching methods, increased responsibilities—particularly financial obligations and higher-level decision-making—and concerns about future career prospects. 29 Depression, a common psychological issue in stressful environments, also affects individuals' ability to perform daily activities. 30

According to previous studies, stress, anxiety, and depression are among the most significant factors associated with insomnia in students. For example, a 2022 study in Oman on 632 students from Sultan Qaboos University reported a significant correlation between insomnia and levels of stress, anxiety, and depression. 11 Similarly, Barakat et al. found a strong association between anxiety, stress, and depression in a study of 980 medical students aged 18–25 at Mansoura University in Egypt. 10 Another study by Choueiry et al. also reported higher levels of anxiety in students with insomnia. 31 Furthermore, Huang et al., using SHAP algorithm analysis, identified depression as one of the most critical factors for accurately classifying individuals into healthy and insomniac groups. 32

It is also worth noting that numerous studies indicate the relationship between disturbed sleep and depression or anxiety is complex and bidirectional. 13 33 In the present study, stress, anxiety, and to a lesser extent, depression, were identified as significant predictors of insomnia.

Other factors associated with insomnia in the present study include bedtime, wake-up time, and the duration of nightly sleep. Studies suggest that the optimal bedtime is between 10:00 PM and 11:00 PM, and the ideal duration for nightly sleep is generally 6 to 7 hours. 1 As shown in the decision tree analysis in Fig. 4 , a nightly sleep duration of less than 7 hours and 15 minutes increases the likelihood of being classified into class 2 (students with insomnia disorder).

As observed in the results, financial, career, and family concerns (which have been identified as the “problem” feature.) are generally associated with insomnia. Previous studies have identified family-related issues as one of the most significant factors exacerbating insomnia. 34 Family problems such as divorce and the death of a spouse are examples of events that can affect sleep and contribute to the increased prevalence of insomnia. 35 Additionally, financial concerns, due to reasons such as heightened stress and anxiety levels or increased working hours, can intensify sleep disorders. 36

Another relevant factor is the prolonged use of digital devices, particularly smartphones. Screen light exposure from smartphones, computers, and tablets is similar to exposure to morning sunlight (which triggers the cortisol awakening response), underscoring the importance of limiting digital device use, particularly close to bedtime. 37 Previous studies report that using a smartphone for more than five hours a day is significantly associated with shorter sleep duration and poorer sleep quality. 11 In the present study, as seen in the SHAP analysis, social media activity and increased hours of digital device usage were associated with a higher likelihood of being classified into class 2.

Although several demographic variables, including age, gender, marital status, residence status, academic major, and body weight, were included in the predictive models, these variables demonstrated relatively lower predictive importance compared with psychological, behavioral, and sleep-related factors. This finding may suggest that insomnia among university students is more strongly associated with modifiable mental health and lifestyle-related factors than with demographic characteristics alone.

The lack of cooperation from some students in completing the questionnaire and participating in the study slowed down the research process. To address this issue and collect accurate data, the sampling period was extended. The study was cross-sectional, and causal relationships cannot be established in such studies. Some questionnaires were excluded from the study due to a significant number of unanswered questions ( *n* =5), and these were not included in the final sample. In this study, as in some similar studies, several factors were self-reported, which may introduce recall bias in the data.

## Conclusion

The present study revealed that the five factors most strongly associated with insomnia among students are fear of not falling asleep, particularly during the late hours of the night, sleep hygiene, anxiety, stress, and depression. Other factors that were also associated with insomnia include bedtime, wake-up time, the duration of nightly sleep, life difficulties such as financial and family problems, and membership and activity in social networks. The findings suggest that machine learning may be useful in identifying patterns associated with insomnia and may help inform future research and personalized intervention strategies. However, due to the cross-sectional design of the study, these findings should be interpreted as associations rather than causal relationships.
